# The limited antegrade subintimal tracking technique to retrieve a trapped rotablator burr: a case report

**DOI:** 10.1093/ehjcr/ytae044

**Published:** 2024-01-25

**Authors:** Annette Maznyczka, Abdul Mozid

**Affiliations:** Swiss Cardiovascular Center, Bern University Hospital, Freiburgstrasse 18, Bern CH-3010, Switzerland; Leeds General Infirmary, Leeds, UK

**Keywords:** Rotational atherectomy, Subintimal tracking, Percutaneous coronary intervention, Case report

## Abstract

**Background:**

Burr entrapment is a rare, but potentially serious complication of rotablation. This report describes the percutaneous options available for Rota burr retrieval.

**Case summary:**

A 62-year-old Caucasian man with stable angina presented for percutaneous coronary intervention. Attempted rotablation with a 1.75 mm burr resulted in Rota burr entrapment, in the heavily calcified proximal right coronary artery. A chronic total occlusion angioplasty technique (limited antegrade subintimal tracking) was successfully used to remove the trapped Rota burr, by enabling subintimal dilatation to externally crush plaque and dislodge the burr. The angioplasty procedure was then completed using the wire that had a short subintimal passage, before re-entering the true lumen.

**Discussion:**

The mechanism for Rota burr entrapment, in this case, was initiating rotablation on the heavily calcified lesion and not more proximal to allow a pecking motion. The learning points are (i) to start the rotablator several millimetres proximal to the actual lesion, and (ii) if unable to wire alongside a trapped Rota burr in the true lumen, then subintimal crossing and balloon dilatation in the subintimal space may work to dislodge the burr.

**Learning points**

- To appreciate risk factors for burr entrapment.
- To become familiar with the percutaneous bailout options to dislodge a trapped burr, in particular the steps involved with limited antegrade subintimal tracking.

## Introduction

In situations where a trapped Rota burr is occlusive of coronary flow and cardiac surgery is not immediately available, successful application of percutaneous bailout options for burr dislodgement may circumvent myocardial damage. Herein, we describe a case where a chronic total occlusion (CTO) angioplasty technique [limited antegrade subintimal tracking (LAST)]^1^ was successfully used to enable the removal of a trapped Rota burr.

## Summary figure

**Figure:**
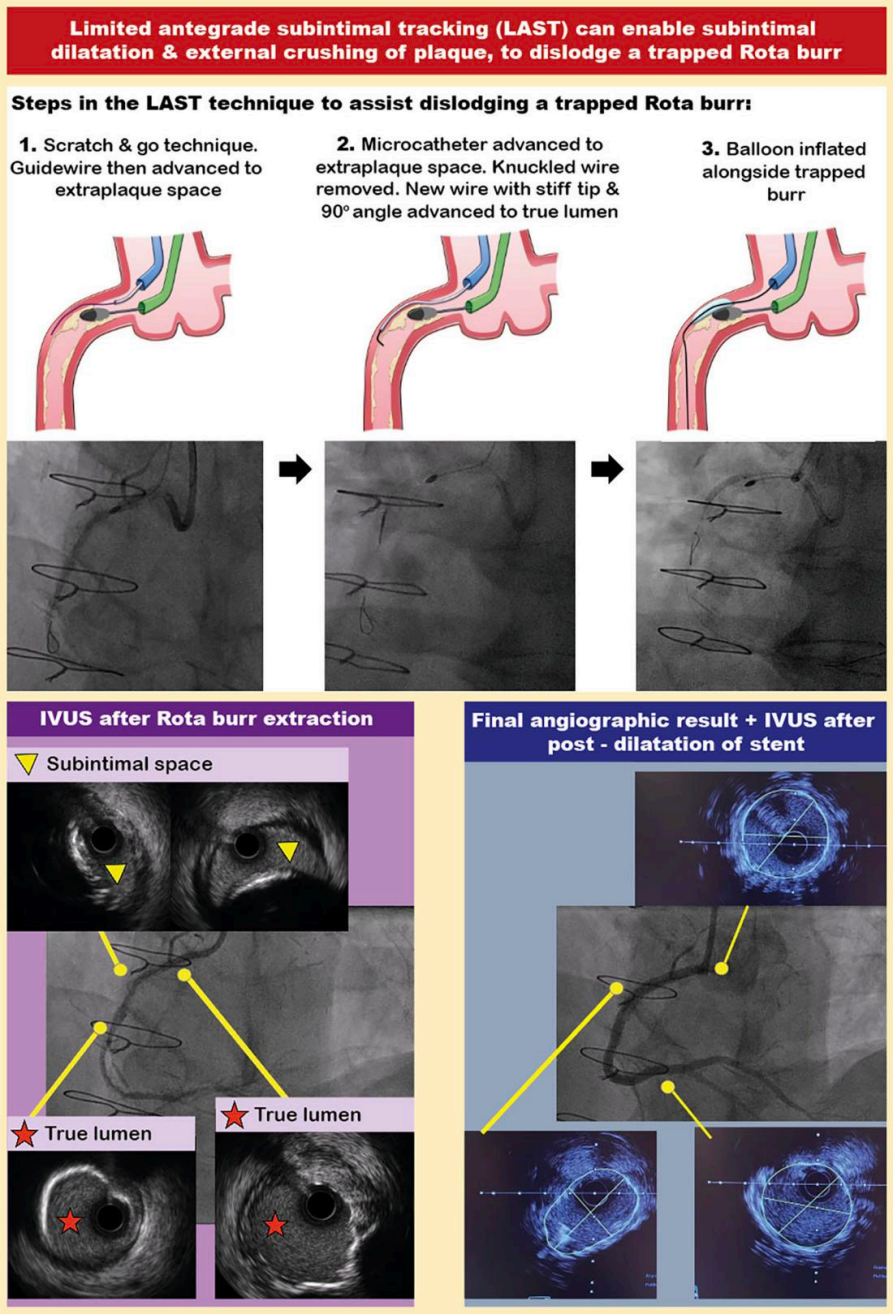


## Case presentation

A 62-year-old Caucasian man had exertional chest pain, consistent with angina. Co-morbidities included diabetes, hypertension, and hypercholesterolaemia. He had coronary bypass graft surgery >10 years previously and was known to have a patent left internal mammary graft to the left anterior descending (LAD) artery and an occluded vein graft to circumflex. He had previous stents to the proximal right coronary artery (RCA), circumflex artery, and vein graft; however, details of those procedures were not available. On examination, heart sounds were normal, and he was clinically euvolaemic. His heart rate was 74 b.p.m., and systolic and diastolic blood pressures were 126 and 78 mmHg, respectively. His symptoms were most likely due to obstructive epicardial coronary artery disease.

**Figure 1:**
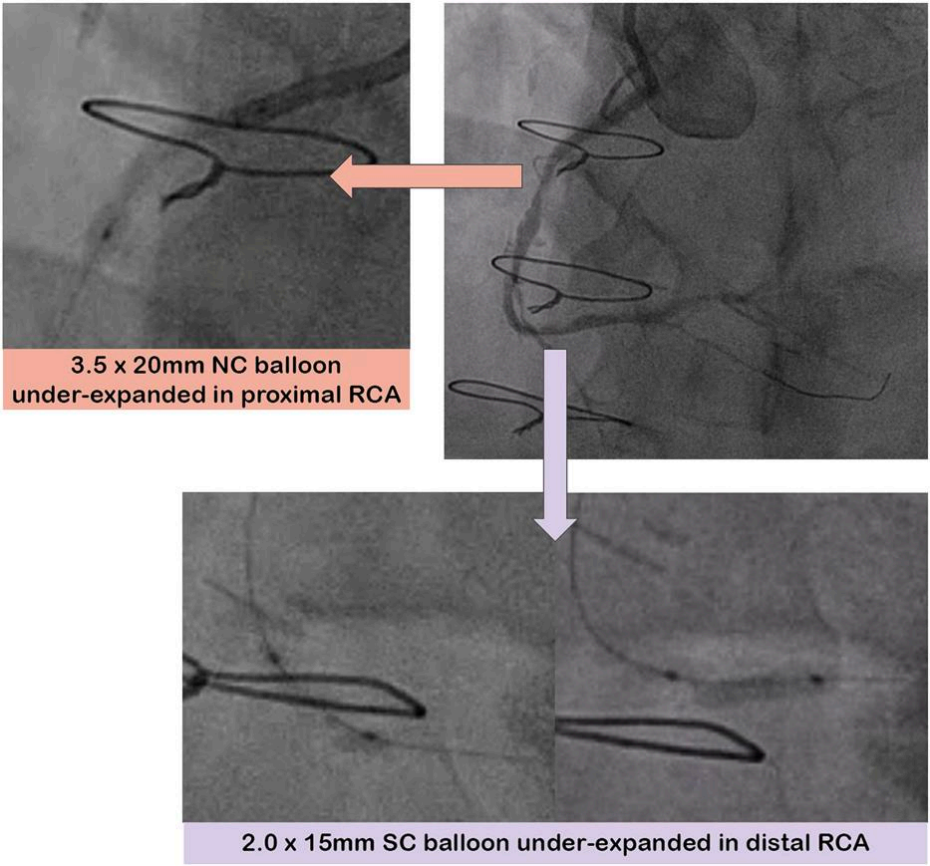
Angiogram and under-expanded balloons in the proximal and distal right coronary artery. NC, non-compliant; RCA, right coronary artery; SC, semi-compliant.

The patient’s renal function was normal (estimated glomerular filtration rate of >90 mL/min/1.73 m^2^), as was his haemoglobin [141 g/L (normal range: 138–172 g/L)]. Echocardiography revealed normal ventricular function and normal valve function. A nuclear myocardial perfusion scan demonstrated inferolateral ischaemia. Angiography revealed moderate distal left main disease and an occluded LAD. There was a circumflex CTO (see Supplementary material online, *Video S1*). The RCA was dominant, with severely calcified ostial, proximal, mid, and distal lesions, as well as severe disease in the posterior descending artery (PDA) and posterolateral (PLV) branches (*Figure 1*; Supplementary material online, *Video S2*).

The non-grafted RCA was chosen as the target to re-vascularize by percutaneous coronary intervention (PCI). We thought this treatment strategy would most likely improve his angina symptoms, given that inferolateral ischaemia was demonstrated non-invasively. We thought that treating the circumflex CTO would have a lower chance of procedural success, because the Japanese CTO score was 3, there were no straightforward retrograde collaterals, and there was a prior unsuccessful attempt at PCI to the circumflex CTO. The patient’s preference was to have PCI, rather than up-titration of anti-anginal medications. The findings from the ORBITA II trial^2^ (placebo-controlled trial of PCI for relief of stable angina) have shown that PCI is very effective for angina symptom improvement, and indeed, the findings from ORBITA II suggest that offering upfront PCI (rather than adding in PCI after up-titration of anti-anginal medications first) is most likely to have a beneficial effect on angina symptoms.

A 7Fr Amplatz 1 guide catheter was used, via a 7Fr left radial artery sheath. With difficulty, a Sion Blue wire (Asahi Intecc, Irvine, CA, USA) passed through the RCA lesions. Initial damping of the pressure trace improved after dilating the RCA ostium with a 3.5 × 20 mm non-compliant (NC) balloon. In the distal RCA, a 2.0 × 15 mm semi-compliant (SC) balloon was under-expanded (*Figure 1*). Therefore, a Turnpike spiral microcatheter (Teleflex, Morrisville, NC, USA) was used to exchange for a Rota floppy wire (Boston Scientific, Marlborough, MA, USA), and rotablation was performed with a 1.5 mm burr (180 000 rpm) from proximal to distal RCA. We then changed to a 1.25 mm burr for further rotablation (180 000 rpm) into the PDA.

We then dilated the RCA from distal to proximal with a 3.5 × 20 mm NC balloon; however, the balloon was under-expanded in the proximal RCA (*Figure 1*). We used a 3.25 × 10 mm cutting balloon (Boston Scientific, Marlborough, MA, USA) and the 3.5 mm NC balloon to higher pressure in this under-expanded segment. However, we were unable to advance 4.0 mm cutting or intravascular lithotripsy balloons (Shockwave Medical, Santa Clara, CA, USA), despite a 7Fr guide extension [Guideliner (Teleflex, Wayne, PA, USA)]. Therefore, we decided to perform rotational atherectomy with a larger burr. On the first attempt with the 1.75 mm burr (180 000 rpm), the burr immediately decelerated and stalled within the heavily calcified lesion and was stuck in the proximal RCA. The suspected reason for Rota burr entrapment, in this case, was initiating rotablation immediately at the site of the calcified lesion (as the guide catheter had been sucked in during Rota advancement) and not more proximal to allow a pecking motion.

**Figure 2:**
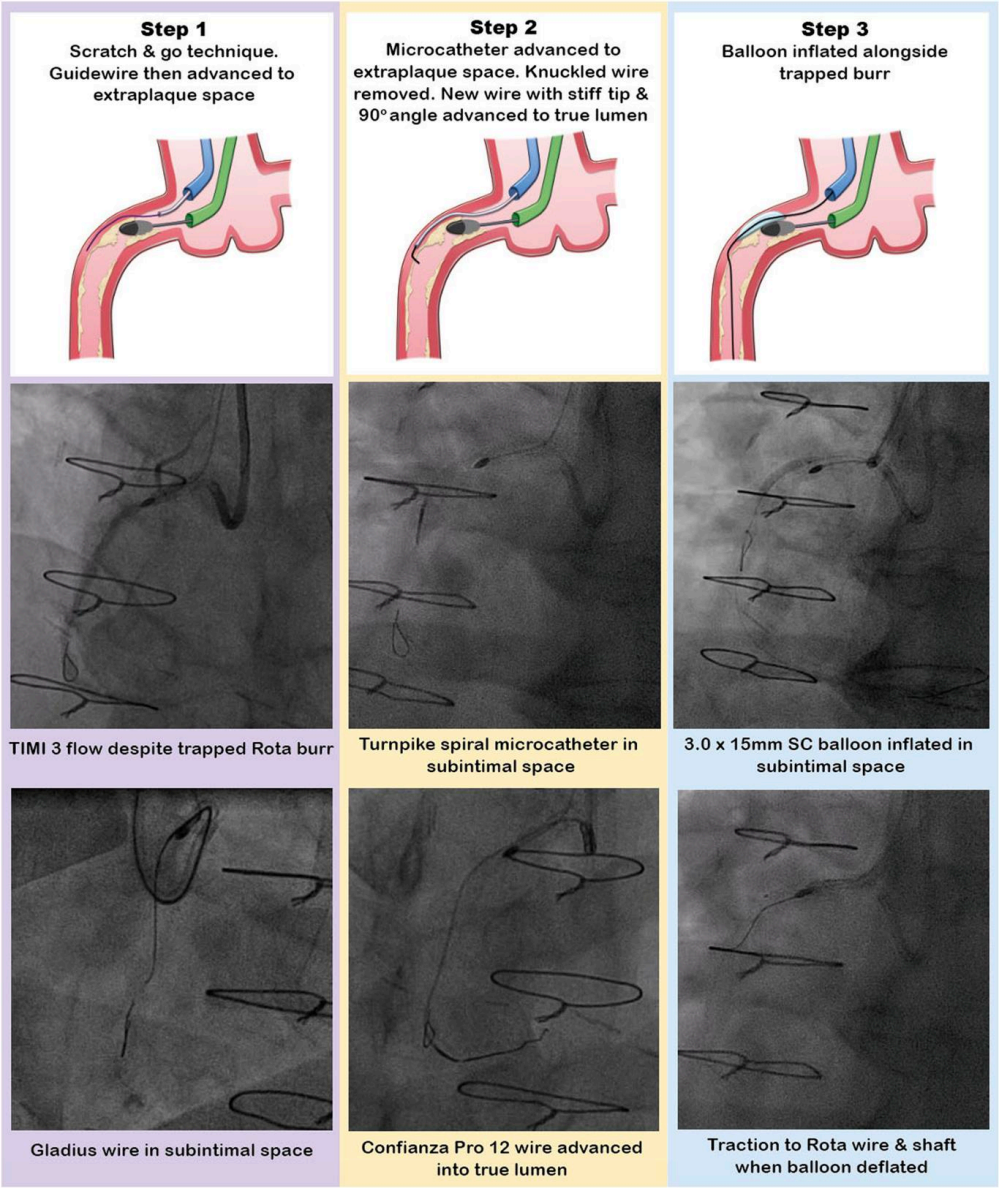
Steps in the limited antegrade subintimal tracking technique. SC, semi-compliant.

Attempts to dislodge the Rota burr by forceful traction on the Rota wire and burr were unsuccessful (see Supplementary material online, *Video S3*). The cardiac surgeon was called but was unavailable to come to the catheter laboratory, so alternative percutaneous options were considered. We advanced a second Amplatz 1 7Fr guide catheter up the right radial artery, for the ping-pong technique.^3^ Using this second guide catheter, we injected contrast and could see that the trapped burr was not occluding the vessel (*Figure 2*). It was not possible to wire alongside the burr in the true lumen; hence, we proceeded to subintimal tracking. We used the ‘scratch and go’ technique, which involved intentionally dissecting the vessel using a hydrophilic, polymer-jacketed Gladius wire (Asahi Intecc, Irvine, CA, USA), to cause a dissection in the vessel immediately proximal to the stuck burr, to access the subintimal space. We achieved this with support from a Turnpike spiral microcatheter. We then advanced the knuckled Gladius wire in the subintimal space beyond the trapped burr. Re-entry into the true lumen was achieved with the LAST technique (*Figure 2*).

For LAST, we used a Turnpike spiral microcatheter to exchange for a non-polymer-jacketed, hydrophilic, stiff Confianza Pro 12 wire (Asahi Intecc, Irvine, CA, USA). A 90° bend at the tip of the Confianza Pro 12 wire was used to steer the wire from the subintimal space into the true lumen. We checked in multiple projections to successfully negotiate the wire into the true lumen and then de-escalated it to a Sion Blue wire. The subintimal space was progressively dilated alongside the trapped burr with 2.0 × 15 mm, and then 3.0 × 15 mm SC balloons. By doing this, we achieved external subintimal crushing of the resistant lesion. After that, forceful traction resulted in the successful extraction of the burr along with the Rota wire (see Supplementary material online, *Video S4*).

**Figure 3:**
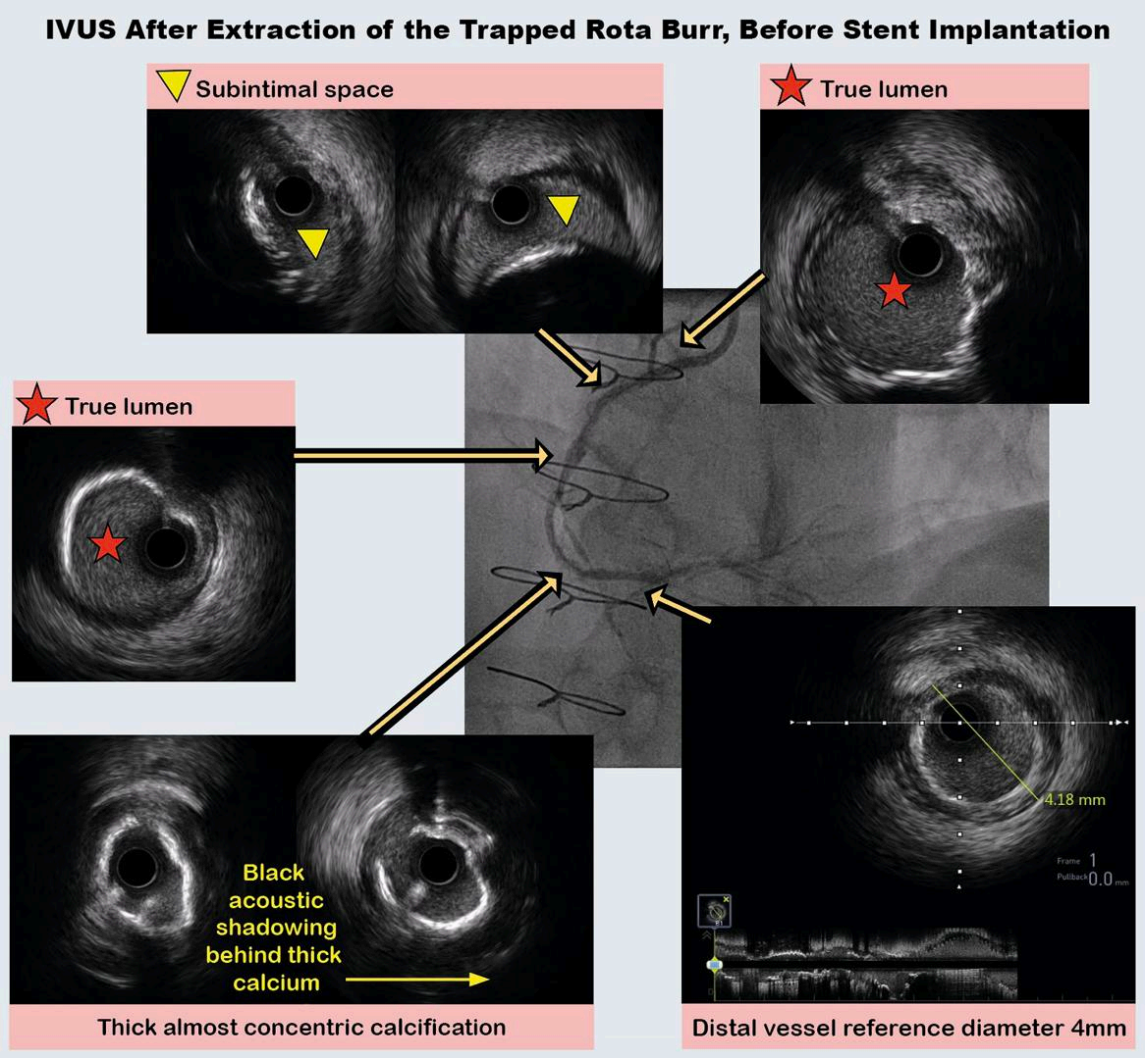
Intravascular ultrasound after burr extraction. IVUS, intravascular ultrasound.

After burr removal, the RCA was dilated with a 3.5 × 20 mm NC balloon, followed by intravascular ultrasound (IVUS) from distal to proximal RCA. Intravascular ultrasound confirmed the wire was in the true lumen from distal to mid-RCA, followed by a short segment of subintimal passage in the proximal RCA, before re-entering the true lumen in the very proximal RCA (*Figure 3*). Intravascular ultrasound demonstrated thick, ≥270° arcs of calcium, with insufficient fractures seen. Eighty pulses from a 4.0 Shockwave intravascular lithotripsy balloon were then delivered from distal to proximal RCA. Two overlapping 3.5 × 48 mm Xience stents (Abbott, Chicago, IL, USA) were deployed from distal to ostial RCA and were post-dilated with a 4.0 × 15 mm NC balloon.

We then turned our attention to the severe PLV-PDA bifurcation disease. Sion Blue wires were advanced to the PDA and PLV, and both branches were dilated with a 2.5 × 15 mm NC balloon. Due to the fact that wiring of both the PDA and PLV branches had been difficult, we did not want to give up the position of these wires prior to stenting. Therefore, we elected to stent the PDA first, with a 2.5 × 33 mm Xience stent, and then crushed the proximal end of the PDA stent with a 2.75 × 15 mm NC balloon on the PLV wire. We then deployed a 2.5 × 48 mm Xience stent in the PLV, which was overlapped proximally with a 2.75 × 23 mm Xience stent from PLV to distal RCA. Once stented, it was easy to re-wire the PDA and perform kissing balloon inflations. If we had felt wiring of the branches would be straightforward, the Culotte technique could have been favoured for treating the PLV-PDA bifurcation, given that the side and main branches had similar diameters, with a narrow (<70°) bifurcation angle. A provisional single stent strategy was not chosen, because the bifurcation disease was complex according to DEFINITION criteria, and in that setting, upfront dual stenting (mainly with double kissing crush) was shown to reduce target lesion failure rates compared with a provisional strategy.^4^ The DEFINITION study defined complex coronary bifurcation lesions (Medina 1,1,1/0,1,1 with side branch diameter of >2.5 mm) as meeting a major criterion and at least two minor criteria.^3^ The major criteria are side branch lesion length of ≥10 mm and side branch lesion diameter stenosis of ≥90% for non-left main bifurcations or ≥70% for left main bifurcations. The minor criteria are as follows: (i) moderate to severe lesion calcification; (ii) multiple lesions; (iii) bifurcation angle <45° or >70°; (iv) main branch reference vessel diameter of <2.5 mm; (v) main branch lesion length of ≥25 mm; and (vi) thrombus containing lesions.^3^

**Figure 4:**
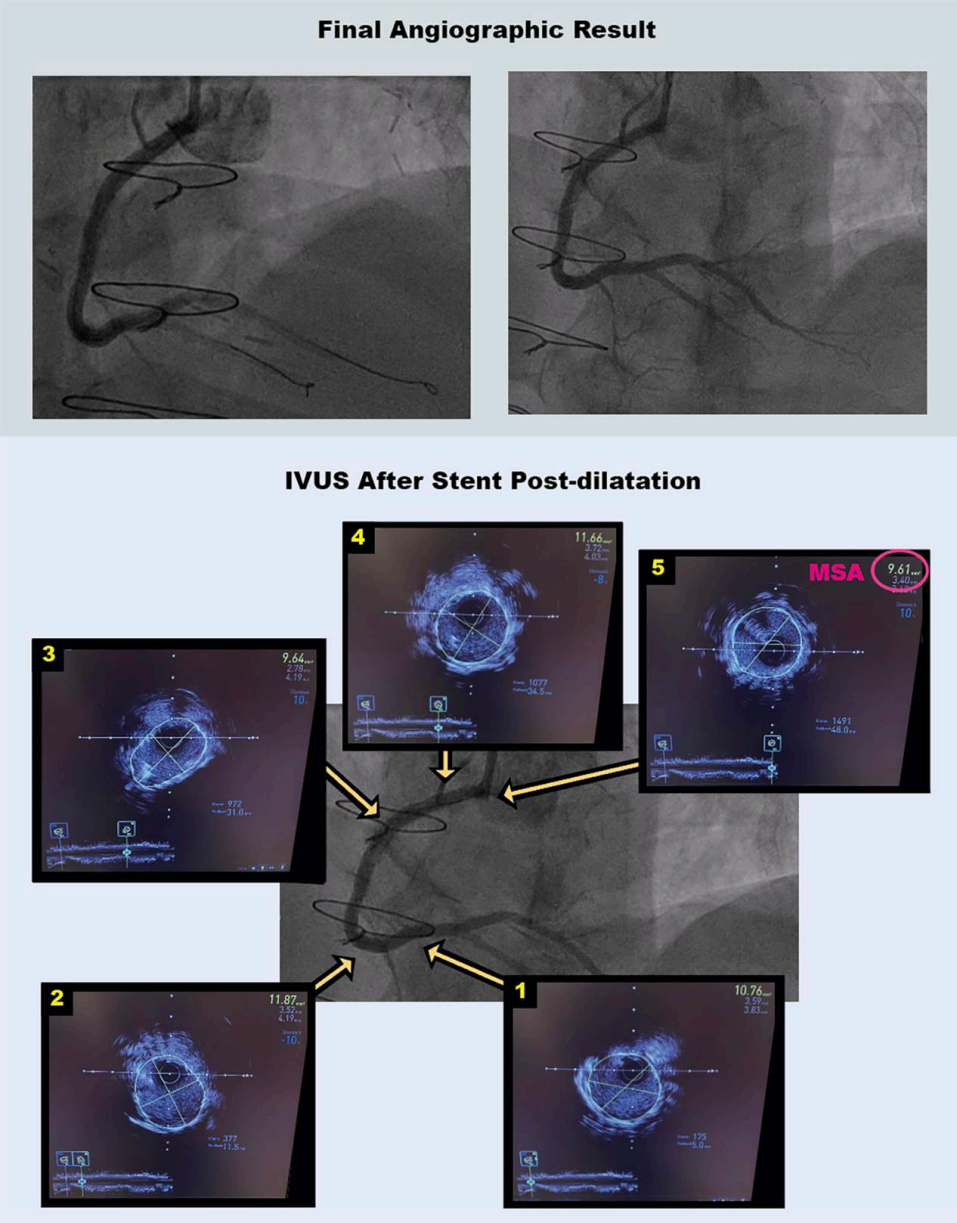
Final angiographic result and intravascular ultrasound post-percutaneous coronary intervention. IVUS, intravascular ultrasound; MSA, minimum stent area.

The final result demonstrated brisk coronary flow (*Figure 4*; Supplementary material online, *Video S5*). Post-PCI IVUS showed that the stent had an elliptical shape in the subintimal segment, but the stent area was 9.6 mm^2^ (minimum stent area) in this segment (*Figure 4*). Furthermore, stent expansion was ≥90% of the distal reference, which is excellent according to IVUS targets in the ULTIMATE trial.^5^

**Figure 5:**
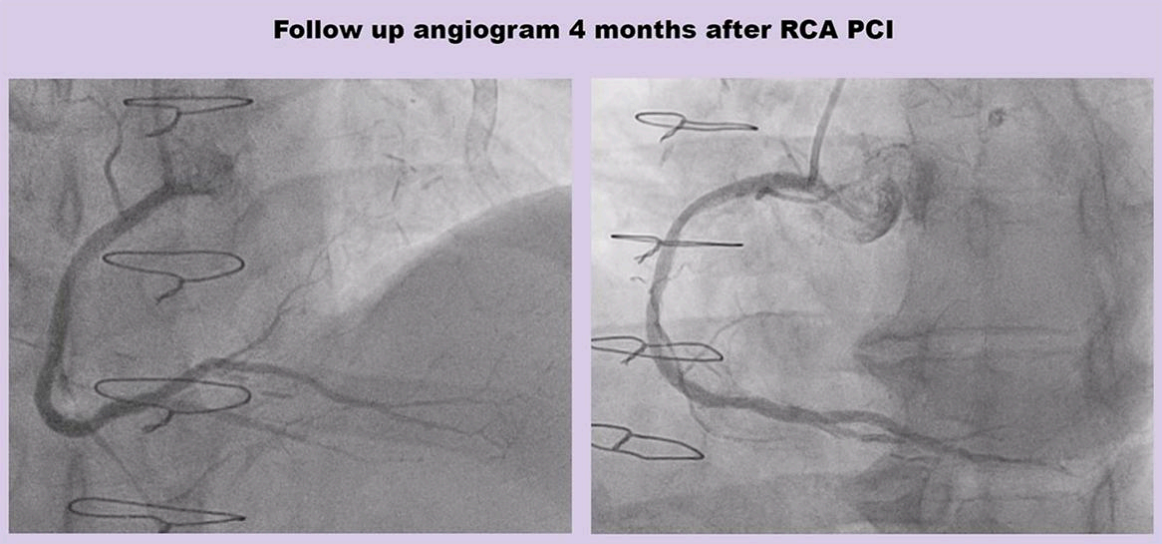
Follow-up angiogram 4 months after percutaneous coronary intervention to right coronary artery. PCI, percutaneous coronary intervention; RCA, right coronary artery.

The patient returned electively 4 months later and had an unsuccessful attempt at PCI to the circumflex CTO. The RCA was re-imaged at that time and demonstrated an excellent a longer-term angiographic result from the PCI procedure (*Figure 5*). He had improvement in his angina symptoms, and any residual symptoms were managed medically.

## Discussion

This case demonstrates the use of the LAST technique to externally crush plaque from the subintimal space, to dislodge a trapped Rota burr. The external subintimal plaque modification technique was originally described for balloon uncrossable CTO lesions.^6^ The LAST technique was developed to minimize extraplaque haematoma by re-entering the true lumen immediately beyond the distal CTO cap, using a wire with a high-gram tip load, for example Confianza Pro 12 (Asahi Intecc, Irvine, CA, USA).^6,7^ Previous reports have described the Subintimal Tracking and Re-entry (STAR) technique, to spontaneously re-enter the true lumen from the subintimal space (usually at a distal bifurcation), using a polymer-jacketed wire with a low-gram tip load, such as Fielder XT (Asahi Intecc, Irvine, CA, USA).^6,8,9^ The potential advantage of the LAST technique, compared with STAR, is that re-entry into the true lumen can usually be more predictable, with a lower risk of side branch loss.^10^ Using IVUS, we demonstrated excellent stent expansion, despite the subintimally stented segment. Importantly, subintimal stenting (with stent in true lumen distal and proximal to the subintimal segment) has been shown to have similar long-term outcomes as stents entirely in the true lumen.^11^ In contrast to previous reports of subintimal tracking to dislodge a stuck Rota burr,^8,9^ our case had a longer-term angiographic follow-up, and this revealed an excellent result (*Figure 5*).

When attempting percutaneous retrieval of an entrapped burr, the first recommended manoeuvre is manual traction to the Rota wire and burr (intracoronary nitrates may relieve coronary spasm for this maneuver^12^). The distal part of the Rota wire has a larger diameter (0.36 mm) than the rest of the wire (0.24 mm), to prevent the burr from passing beyond the wire tip. Therefore, manual traction on the Rota wire may dislodge the burr, so that both can be removed *en bloc*. However, extreme force during manual traction risks rota shaft fracture, iatrogenic guide catheter-induced dissections, and vessel perforation.

If simple manual traction is unsuccessful, percutaneous methods to increase local withdrawal force may be attempted. One technique is to cut the rota shaft immediately distal to the rota connector, remove the outer plastic cover of the rota shaft, and then advance a guide extension, or child-in-mother catheter over the rota shaft,^13^ as close as possible to the trapped burr. Simultaneous traction on the rota shaft and counter traction on the child catheter can increase withdrawal force. Another technique is to encircle a Goose Neck snare (Medtronic, Minneapolis, MN, USA) over the rota shaft, advance the snare close to the trapped burr, tighten it, and apply manual traction to the snare and Rota shaft.^14^ The third option is the ping-pong technique to advance a second wire alongside the trapped burr and then inflate a balloon alongside the burr to dislodge it.

Tips to reduce the risk of Rota burr entrapment include (i) avoiding decelerations >5000 rpm by using gentle pecking motion and shorter ablation runs (<20 s) and using higher rotablation speeds and (ii) avoiding rotablation through a recently implanted stent or in severely tortuous vessels.

## Conclusion

In conclusion, the main learning point from this case is to make sure the rotablator is started several millimetres proximal to the lesion, to allow a pecking motion. In this case, the rotablator was started on the lesion, which was the suspected reason for burr entrapment. The second learning point is that when unable to wire alongside a trapped Rota burr to dilate within the true lumen, then subintimal crossing and external modification from the subintimal space can work to dislodge a trapped Rota burr.

## Supplementary Material

ytae044_Supplementary_Data

## Data Availability

All available data relevant to this case are presented within the manuscript.

## Lead author biography

Dr Annette Maznyczka was awarded CCT in Cardiology in 2022. She completed complex PCI training in Portsmouth and Leeds and is now undergoing valve intervention training in Bern, Switzerland, funded by the EAPCI. Annette is enthusiastic about clinical research and education and has completed a PhD in invasive coronary physiology and an MSc in medical education.

## Supplementary material

**Consent:** Written consent for the submission and publication of this case report has been obtained from the patient in line with COPE guidance.

**Funding:** There was no funding for this case report.
